# Biodiversity loss decreases parasite diversity: theory and patterns

**DOI:** 10.1098/rstb.2012.0110

**Published:** 2012-10-19

**Authors:** Kevin D. Lafferty

**Affiliations:** Western Ecological Research Center, US Geological Survey, c/o Marine Science Institute, University of California, Santa Barbara, CA 93106, USA

**Keywords:** coextinction, food web, parasite, biodiversity loss

## Abstract

Past models have suggested host–parasite coextinction could lead to linear, or concave down relationships between free-living species richness and parasite richness. I explored several models for the relationship between parasite richness and biodiversity loss. Life cycle complexity, low generality of parasites and sensitivity of hosts reduced the robustness of parasite species to the loss of free-living species diversity. Food-web complexity and the ordering of extinctions altered these relationships in unpredictable ways. Each disassembly of a food web resulted in a unique relationship between parasite richness and the richness of free-living species, because the extinction trajectory of parasites was sensitive to the order of extinctions of free-living species. However, the average of many disassemblies tended to approximate an analytical model. Parasites of specialist hosts and hosts higher on food chains were more likely to go extinct in food-web models. Furthermore, correlated extinctions between hosts and parasites (e.g. if parasites share a host with a specialist predator) led to steeper declines in parasite richness with biodiversity loss. In empirical food webs with random removals of free-living species, the relationship between free-living species richness and parasite richness was, on average, quasi-linear, suggesting biodiversity loss reduces parasite diversity more than previously thought.

## 1. Introduction

How should parasites respond to biodiversity loss? The field of conservation biology often views infectious organisms as a sign of imbalance and emphasizes how stressors such as climate change [1] and loss of biodiversity [2] might promote infectious disease. Indeed, some studies find that decreased diversity of non-competent hosts can increase transmission of a pathogen to species of concern, the most cited examples being West Nile virus [3], and Lyme disease [4]. Such a reduction in disease risk to human populations is heralded as an ecosystem service that can be used to market the value of biodiversity [5].

A broader perspective recognizes that parasites can decline with biodiversity loss, and parasites could make up the unseen majority of species extinctions [6–10]. Many parasites depend on complex and functioning ecosystems [11–17]. For instance, each stage in a parasite life cycle requires at least one host species. Hence, medical geographers consider how distributions of ‘vector’ hosts set the distributions of human infectious diseases. Mosquito distributions limit the reach of malaria, tsetse flies make sleeping sickness possible, black flies transmit river blindness, schistosomiasis requires certain snails, Chagas disease is absent without kissing bugs and leishmaniasis depends on sand flies. For these reasons, vector control (a type of biodiversity reduction) is a key strategy for controlling infectious disease. The link between host and parasite distributions applies to non-medically important parasites as well. For example, the trematode *Pleurogonius malaclemys* infects snails only in the presence of the endangered diamondback terrapin, the sole final host for the trematode [18]. When a diamondback terrapin population is extirpated, it takes its host-specific parasites with it. In this study, I examined factors that influence how parasite richness declines with biodiversity loss.

Some studies have used parasite species lists from different hosts or locations to make predictions about how free-living species diversity relates to parasite diversity. For instance, the numbers of human parasite species and free-living species at a location decrease in richness with latitude, suggesting that either the same geographical factors affect both groups or that reductions in free-living species diversity reduce parasite diversity [19]. Similarly, countries with a higher diversity of birds and mammals have a higher diversity of human parasites [20], though this could be a spurious correlation driven by increases in sampling effort with country size. Not surprisingly, locations in North America with many carnivore species have longer combined carnivore parasite species lists, leading to a strong positive correlation between carnivore diversity and the estimated diversity of carnivore parasites [21]. Such ‘list’ studies are instructive, but their patterns have many alternative explanations, including sampling artefacts.

A few field observations have linked the diversity of parasite communities in a single host to the diversity of other hosts in the system. Most notably, the richness of trematode communities in snails increases with the diversity of birds that are final hosts for the worms [22]. Different final hosts have different diets, and this exposes them to different parasites, leading to distinct parasite ‘signatures’ in the snail population [23,24]. The diversity of invertebrates (many of which are second-intermediate hosts for the trematodes) also correlates with the diversity of trematodes in snails; however, spatial patterns can break down for mobile hosts (such as fishes) that tend to homogenize associations in snail parasites [12]. These examples suggest that efforts to protect free-living biodiversity can also increase parasite diversity. For coral reefs [25], the rocky intertidal [26], lake shores [27] and estuaries [14], sites protected from human disturbance have more parasites than impacted sites.

**Figure 1.**
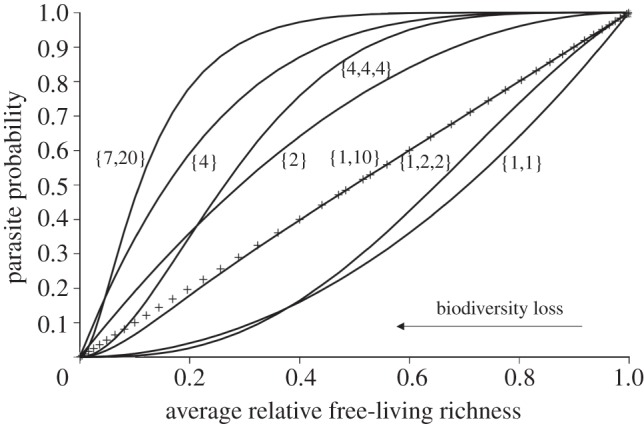
Life stages and generality combine to affect the robustness of parasites to secondary extinction. Plotted are the hypothetical standardized relationships between free-living species diversity and parasite probability for parasites (model (2.3*e*)). The axes are standardized relative measures of free-living species diversity and the probability that a parasite will be present. Biodiversity is lost from the system by moving from right to left along the horizontal axis (arrow). The number of life stages is the length of the set in brackets and hosts per stage are indicated as integers. For example, the line to the far left indicates a parasite with a first stage that can use seven hosts and a second stage that can use 20 hosts. The straight line indicated by the ‘+’ symbols is for a host-specific, single-stage parasite.

If one were to plot parasite richness against free-living species richness, what would the shape of the relationship be? Imagine that an intact system with 100 per cent of its parasite species and 100 per cent of its free-living species occupies the upper right-hand corner {1, 1} of a standardized richness–richness plot such as in figure 1. Clearly, all parasites must go extinct when there are no hosts, sending the system to {0, 0}. Although the endpoints of the relationship between parasite and free-living species richness are obvious, the path between these endpoints is not easy to predict. A concave down relationship would suggest that parasites are robust to reductions in free-living species diversity (here, ‘robustness’ is an inverse measure of secondary extinction risk of species or groups of species). By contrast, a concave up relationship would show that parasites are sensitive to biodiversity loss. A sigmoid relationship would indicate a threshold in free-living species diversity loss that, once crossed, leads to a rapid collapse of the parasite community. Might the shape of the relationship vary among systems? If so, what factors affect the relationship?

I start by investigating a series of analytical models (table 1) that relate parasite diversity to free-living species diversity. The parasites in these models vary in the number of life stages and host specificity per life stage, whereas the hosts vary in extinction order (random, non-random or fixed). The models can be made for single parasite species, or parametrized for an average parasite species, or averaged across parasite species. This reveals classic modelling trade-offs. Mean-field approximations (modelling the average parasite species) take less data and computation, but require more assumptions, than do models that track every stage of every parasite species. A key assumption of current analytical models is that hosts do not suffer secondary extinctions when they lose their resources. Because hosts are embedded in food webs where secondary extinctions might be important, I adapt food-web disassembly to model how parasite diversity relates to free-living species diversity. This is done for hypothetical and empirical food webs. Finally, the patterns produced by the different models are summarized and compared.

**Table 1.** Types of analytical models developed.

model	host extinction	life cycle	hosts per stage	parasite spp.
(2.1*a*,*b*)	random	simple	specialist	single
(2.1*c*)	random	simple	specialist	multiple
(2.2*a*)	random	simple	generalist	single
(2.2*b*)	random	simple	average	multiple
(2.2*c*)	random	simple	generalist	multiple
(2.3*a*)	random	complex	average	single
(2.3*b*)	random	complex	corrected average	single
(2.3*c*)	random	average	corrected average	multiple
(2.3*d*)	random	complex	corrected average	multiple
(2.3*e*)	random	complex	generalist	single
(2.3*f*)	random	complex	generalist	multiple
(2.4*a*)	variable	simple	specialist	single
(2.4*b*)	variable	complex	generalist	single
(2.4*c*)	variable	complex	generalist	multiple
(2.5*a*)	ordered	complex	generalist	single
(2.5*b*)	ordered	complex	generalist	multiple

## 2. Methods

### (a) Background

To better understand the effect of free-living species richness on parasite richness, I applied probability theory and simulation modelling to hypothetical and real communities. As a conceptual framework, I assumed a contained system, such as an island or a lake, where species could be extirpated but could not recolonize. The units tracked were parasite species and free-living species, including non-hosts. To be able to compare different systems in a common currency, I expressed the richness of free-living species as a proportion (0–1) relative to the maximum (initial) richness of free-living species in the system. Parasite species richness was expressed on the same relative scale (e.g. proportion of the maximum number of parasite species). Individuals within species were not tracked, so there was no measure of abundance. However, I did specify life stages within species. For simplification, and owing to the focus of this review, I did not consider life stages of free-living species in hypothetical food webs (though some free-living species of empirical food webs did indeed have discrete life stages [28]). I also assumed that parasites did not affect free-living species diversity.

I assumed that an outside force (biodiversity loss) directly removed free-living species in a (usually) random sequence, otherwise called ‘primary extinction’ (see later text). Parasite species were not directly removed (e.g. I assume no targeted parasite-eradication effort such as the one to eliminate smallpox). Parasites that could no longer complete their life cycles experienced a secondary extinction. This form of secondary extinction is a conservative approach for determining the ‘robustness’ of communities to perturbations such as biodiversity loss [29].

I first developed simple analytical models of the system mentioned earlier to generate predictions about how the generality of parasites, life-stage complexity and differential extinction risk of hosts would affect the relationship between free-living species diversity and parasite diversity. I then used food-web disassembly models to investigate these predictions in hypothetical and empirical food webs.

### (b) Analytical models

Here, I step through the analytical models from the least data intensive to the most complex, noting the assumptions and limitations of each. In general, I first build models for individual species and then adapt individual species models to communities of parasites. The more promising of these models are then given names and compared in following sections.

#### (i) Simple host-specific models

If a parasite present in a system is host-specific (generality *g* = 1), and has a single life stage, *s*, then the probability it will remain in the system is equal to the probability its host will remain in the system (assuming the system starts with the parasite being present), or2.1a
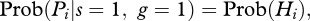
where *P_i_* is the presence of a parasite species *i* in the system and *H_i_* is the presence of the required host for *P_i_* (the symbol ‘|’ specifies the model assumes the statements to the right). Assuming hosts and non-hosts have the same probability of being independently lost from the system, we can generalize (2.1*a*) to2.1b
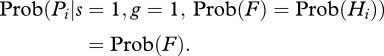
At the community level, the relative species richness (0–1) of parasites (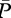) and relative species richness (0–1) of free-living species (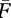) are equal to the average probability of occurrence, so 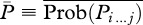 and 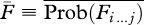. Equation (2.1*b*), expressed in terms of relative species richness, is, therefore2.1c
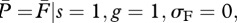
where *σ*_F_ is the standard deviation of the probability of each free-living species being present. When plotted as relative richness of parasites versus free-living species, there is a straight line (the 1 : 1 line) from {1, 1} to {0, 0}, consistent with the suggestion that the number of threatened parasite species is a linear function of the number of threatened hosts [9]. This is the simplest of all models and its key assumption of strict host specificity is not realistic for most parasite communities.

#### (ii) Generality

Because generalists are more robust to secondary extinction than are specialists [30,31], the extent of parasite generality will affect the persistence of parasites in the face of biodiversity loss. As an example, one louse species escaped global coextinction with the passenger pigeon because it also infected other pigeons [32]. A community composed of generalist parasite species should maintain its richness even after the random removal of several free-living species. Koh *et al*. [8] found the association between parasite richness and host richness to be increasingly concave down as the average number of hosts per parasite increases.

Including generality leads to a sampling without replacement problem. Koh *et al.* [8] provide an analytical approximation for datasets with single-stage parasites. Binomial probability models are far simpler, but will overestimate the probability of a parasite for concave down trajectories and underestimate it for concave up trajectories. However, for sample sizes of more than 25 free-living species, simple binomial models make a good approximation. All the datasets analysed later had sample sizes above this threshold, so sample size corrections were not used. Another complication of generality is that the parasite can persist even if only one of its hosts remains. The probability that this occurs is the complement of not having a host, or 
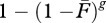
. For a single parasite species, again assuming equal extinction probabilities among free-living species, the simplest binomial approximation is2.2a
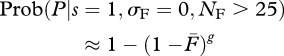
where *g* (generality) is the number of hosts for a parasite with a single stage (or of a single stage of a parasite). I used this model to illustrate the effect of generality for single-stage parasites with two and four hosts (figure 1). A similarly simple model for a parasite community requires the assumption that parasites do not differ in generality.2.2b
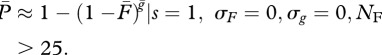
However, if there is variation in generality among parasite species, estimating the relative richness of the parasite community requires averaging among parasite species.2.2c
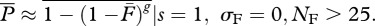
Koh *et al*. [8], however, provide an analytical approximation that uses the distribution of *g* among species.

#### (iii) Life stages

Many parasites require more than one type of host to complete their life cycle (e.g. malaria requires a vertebrate and a mosquito). For this reason, parasites with complex life cycles should be more sensitive to reductions in free-living biodiversity [28,33,34], depending on the generality of each life stage. However, the effect of life stages on the relationship between parasite diversity and free-living species diversity has not yet been investigated.

Now, for a parasite species, we can write2.3a
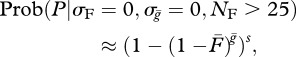
where *g* now refers to the generality of a parasite stage, not a parasite species.

If there is variation in generality among stages within a parasite (*σ_g_* > 0), using the arithmetic mean of the generality per stage for *g* can greatly overestimate the robustness of a parasite because, as shown later, stages with low generality have a disproportionate effect of robustness. The geometric mean is a better approximation, but still underestimates the robustness of a parasite if there is variation in generality among stages and this underestimate increases with *F*. To provide a correction to the underestimate, I considered2.3a
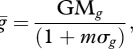
where GM*_g_* is the geometric mean of the generality among stages within a species. Here, *m* is a slope that indicates how the average standard deviation of generality within a species (*σ_g_*) influences robustness as a function of *F*. For values of *F* from 0 to 1, I then solved for the *m* that best fit the hypothesis for lists of integer exponents that varied in their standard deviations. This resulted in a list of best-fit slopes for each value of *F*. The best fit to these data was *m* = 0.2*F*, so that2.3b
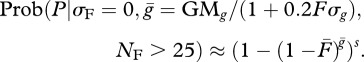
Assuming no variation in the number of stages per parasite species, for a parasite community, the ‘average parasite model’ would be2.3c
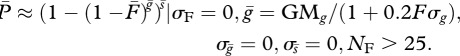


However, variation in the number of stages per species and the average number of hosts per stage could lead to considerable error in the average parasite model (2.3*c*). A more data-intensive version (but with fewer assumptions) is the average of equation (2.3*b*) across parasite species, leading to the ‘average stage model’2.3d
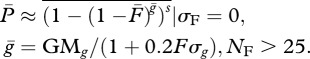


For a single parasite species, an even more precise model, with even fewer assumptions would explicitly account for variation in generality among parasite stages, resulting in2.3e
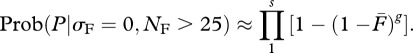


To illustrate the combined effect of life stages and generality, I calculated the trajectories of multi-stage parasites and single-stage parasites for equation (2.3*e*) (figure 1). The parasites differed in the distribution of generality among stages. For example, the first parasite used seven host species in one stage and 20 host species in a second stage. The coding for the first parasite was {7,20} and the other multistage parasites were {4,4,4}, {1,10}, {1,1} and {1,2,2}.

Averaging across parasite species gives the most precise (and data intensive) measure of robustness for a parasite community, leading to the ‘variable parasite model’.2.3f
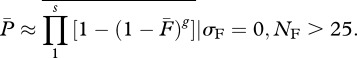


Note that this model has the fewest assumptions of the models so far but requires information about the number of hosts used by each stage of each parasite.

#### (iv) Variable extinction risk among free-living species

**Figure 2.**
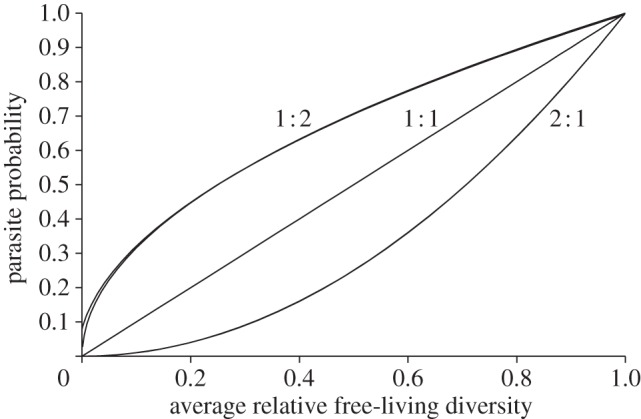
The relative extinction risk of hosts affects how parasite richness declines with free-living species richness. Here, host species differ from non-hosts in how likely they are to be removed from the system during each draw (model (2.4*a*)). Trajectories were calculated for weights of 2 : 1, 1 : 1 and 1 : 2, representing extinction probabilities of hosts : non-hosts (next to the appropriate trajectory).

Earlier studies have assumed that the probability of host extinction is uniform and independent of the number of parasite species per host species. This might not be the case. The rare and endangered species most likely to suffer primary extinctions might have few parasites because some parasite species will have been lost when the host became rare [35]. Under this scenario, if populations had already been depleted, parasite richness might thereafter appear to decline relatively slowly with biodiversity loss. Alternatively, large species might be more likely to suffer primary extinctions [36] and tend to host more parasite species [37]. This would decrease the robustness of parasites to biodiversity loss. Free-living species also vary in their risk of secondary extinction. In particular, specialists and top predators are more likely to go extinct owing to lack of resources during biodiversity loss. Because basal taxa have no risk of secondary extinction, plant parasites should be more robust to biodiversity loss than parasites of top predators. This is important because parasite diversity can increase with host trophic level [38], potentially leading to a negative association between parasitism and host robustness to secondary extinction. Pushing the pattern in the opposite direction is that parasites are more diverse in hosts with broad diets [37,39], which are also less sensitive to secondary extinction [36]. Finally, if parasites make hosts more extinction-prone [40], hosts with lots of parasites may be more likely to go extinct. Although there is no consensus on how sensitive hosts should be to extinction relative to non-hosts, it seems useful to be able to accommodate variation in extinction rates among free-living species. As a starting point, equation (2.1*c*) can be modified to2.4a
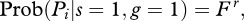
where *r* is the risk of a host being absent from the system relative to all other free-living species. If *r* is high, the host is sensitive to extinction, and the parasite will be more likely to suffer a secondary extinction. Obviously, the extent parasites decline with free-living biodiversity loss depends on how extinction-prone their hosts are relative to non-hosts. As an example, I used model (2.4*a*) to calculate trajectories for situations where the single host of a parasite had equal, twice or half the chance of primary extinction of other free-living species (figure 2). A more general model for a single parasite with stages and multiple hosts is2.4b
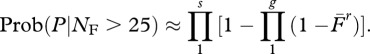
In addition, for a parasite community, the ‘variable host and parasite model’ is2.4c
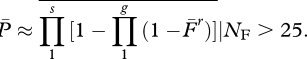

This model has the fewest assumptions of all (and requires the most detailed input). For illustration purposes, the variable host and parasite model was run for an empirical food web with hypothesized variation in extinction risk (see later text).

#### (v) Non-probabilistic models

An alternative non-probabilistic model can be used for cases where an extinction order, *O_i_*, of the hosts and non-hosts can be hypothesized. In other words, suppose free-living species will go extinct in order of their rarity or threat level (e.g. host *q* is the 14th free-living species to go extinct, and host *k* is the 44th free-living species to go extinct) [41]. To simplify the calculations, simultaneous extinctions of free-living species do not occur (though simultaneous extinctions of parasite species are possible). With this information, we should be able to discern the extinction order of the parasites in the system. Extinction order relates to the previous probability models, because the expected order of extinction of a species is inversely related to its probability of extinction. Estimating extinction order for a parasite species requires the same information as model (2.4*c*), but uses actual extinction orders of hosts instead of relative extinction risk. For each parasite, one first estimates the extinction order of the hosts for each stage. Because the stage can persist until its last host goes extinct, the extinction order of a parasite, *O*_p_, is the minimum of the maximum host extinction orders for each parasite stage2.5a
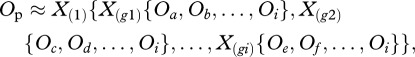
where *O*_p_ is the extinction order of parasite, *X*_(*gi*)_ is the maximum of the extinction orders of hosts (*a* through *i*) for a parasite stage *i*, and *X*_(1)_ is the minimum of the maximum host extinctions across all stages of the parasite. Equation (2.5*a*) is repeated for each of the *N*_p_ parasite species in the system. From the set of *N*_p_ parasite extinction orders, it is possible to calculate how relative parasite richness declines with biodiversity loss. After the *n*th free-living extinction, the proportion of parasites that have an extinction order > *n* will still be present in the system. The set of points representing the relative parasite richness corresponding to a value of relative free-living species richness is the ‘variable host and parasite extinction order model’2.5b
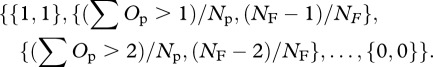


This model is not particularly useful when there is a lot of uncertainty in the extinction order of hosts (making a probabilistic model such as (2.4*c*) more appropriate).

### (c) Food-web models

The analytical models mentioned above do not account for connections among hosts or between hosts and non-hosts. These connections create network structures that can lead to variation in the risk of secondary extinction among free-living species. Furthermore, food webs can lead to correlated patterns of extinction in hosts that, as I will illustrate, can alter the trajectory of extinction for parasites. For this reason, I used a modified form of robustness analysis to incorporate food-web structure into the relationship between parasite and free-living species richness. Robustness analysis considers the presence–absence of species in a food web, focusing on secondary extinctions that result from resource loss [30,31]. It takes the concept of secondary extinctions of parasites that have been the key to the analytical models mentioned above and extends them to free-living species as well. Instead of using binomial analytical models, however, robustness of food webs is performed by computation. This topological approach to simulating disassembly of ecological communities requires few assumptions about how species interact, and thus allows analyses of complex species dependencies not amenable to dynamical modelling [29]. However, food-web disassembly requires a large amount of information about a system and it takes many disassemblies to arrive at an expected result if extinction order is not set.

I created a program in Mathematicata to disassemble large matrices multiple times. For each iteration, the program randomly removed one free-living species from a topological food web as a way to simulate biodiversity loss. Any species (parasitic or free-living) left without resources went secondarily extinct. I then calculated the number of species remaining in the food web separately for parasites and free-living species. This process continued until no species remained. The trajectory of parasite species loss during a single iteration depended on the order in which free-living species were removed during a disassembly, and there are up to *N*! sequences of species removals (where *N* is the number of free-living species). Unless otherwise indicated, extinction order was randomized, and the disassembly was repeated 500 times (known from past work to give good average estimates of disassemblies [42]). Averaging the 500 simulations gave a prediction for the relationship between average relative parasite richness and relative host richness.

#### (i) Effects of food-web topology

To illustrate how food-web structure can affect the relationship between parasite and free-living species diversity, I constructed simple food webs with five free-living species and two parasite species. In all cases, the parasites had a single host, but the structure of their food webs varied remarkably. In the first food web, parasites were independent, and the free-living species were all basal, so no free-living species suffered a secondary extinction. This was the same assumption of the analytical models mentioned above. I then changed these assumptions by making a second simple food web in which the three non-hosts formed a food chain, increasing the risk of secondary extinction of non-hosts compared with hosts. In a third system, the two hosts were consumers; each specialized on a separate basal species. A fourth food web consisted of two hosts sharing the same basal resource so that their fates were no longer independent. I then used the disassembly approach described earlier to calculate the average extinction sequence for the parasite community. To illustrate the effect of correlated extinction between parasites and consumers, I constructed two simple food webs with 10 free-living species and one parasite species. In all cases, the parasites had a single host, but the structure of their food webs varied only in that the parasite shared or did not share its host with other consumers.

#### (ii) Empirical food webs

I applied the disassembly approach to nine empirical food webs with parasites. These were a New Jersey stream, Muskingham Brook [43], the pelagic web of an Arctic lake, Takvatn [44], and seven estuaries: the Ythan River Estuary [45], Otago Harbour [46], Flensburg Estuary [47], Sylt Estuary [48], Carpinteria Salt Marsh, Estero de Punta Banda and Bahía San Quintín [49]. I examined and explained the variation in the shapes of the relationship between free-living and parasite diversity across the nine systems and compared these with predictions from analytical models. I considered that detritus was the last resource to go ‘extinct’. Otherwise, initially, all species were assumed to have the same risk of secondary extinction.

To explain variation in the robustness of single parasite species to secondary extinction, I calculated the relative order of extinction during disassembly for each species. Parasites that went extinct later, on average, than other species in the food web were assumed to be more robust to secondary extinction [33]. For this measure, relative order = 1 roughly corresponds to a linear, less than 1 to a concave up, and more than 1 to a concave down trajectory for parasite richness with biodiversity loss. I also tracked the following statistics for each parasite: number of stages, mean and standard deviation of the number of hosts per stage and the average relative order of host extinction, nested within parasite stage. I predicted that the robustness of a parasite to secondary extinction would increase with increasing generality per life stage and with the robustness of its hosts. I also predicted a parasite's robustness would decrease with the average number (and variation) of life stages. To determine which factors explained parasite robustness, I used a generalized linear model (GLM), considering food web as a random effect.

I asked similar questions about the parasite community as I asked for parasite species. For each food web, I measured the robustness of the parasite community to biodiversity loss as the average relative order of extinction for the parasite species in comparison with free-living species. As before, I predicted that parasite robustness would increase with average parasite generality, decrease with the average number of parasite stages, and increase with the robustness of hosts to secondary extinctions. I again used a GLM, considering each food web as a replicate.

Finally, I considered non-random extinction probabilities for the Carpinteria Salt Marsh food web, because this was a system where I had enough information to propose hypotheses for the relative extinction risk among free-living species. The first hypothesis was that extinction probability decreased with biomass density under the assumption that rare species (controlling for the effects of body size on density) were more likely to be extirpated from a system. The second hypothesis was that the risk of extinction decreased with the frequency that a species was present in three similar estuaries [49]. For instance, species that occurred only in one of the three sites were assumed to be three times more likely to go extinct than species found in all three sites. Some nodes were aggregated taxa such as phytoplankton. I assumed these had a low (one-fifth) rate of extinction relative to single-species nodes. One snail, *Cerithidea californica*, was known to have been extirpated from several sites in other parts of its range [50], and it was assumed to have a 10-fold rate of extinction risk. The variable host and parasite model (2.4*c*) and the variable host and parasite extinction order model (2.5*b*) was run for each list and the results compared with uniform extinction risk.

#### (iii) Evaluating model performance

Ideally, an analytical model should be simple, but fit the data well. To compare the performance of the various analytical models, I used the nine food-web models mentioned earlier as a benchmark. For a particular set of inputs, I calculated *P* and average *P* for a food-web model and the analytical models. For a range of biodiversity loss from 0 to 100 per cent, I calculated the average absolute deviation of each analytical model from the food web model. I also noted if there was a bias in a particular direction. Deviations were calculated at the level of individual parasites and for the parasite community. In addition, to illustrate model fit, I plotted the predictions of the three analytical models and the food-web model for a lake and stream food web.

## 3. Results

### (a) Analytical models

#### (i) Generality of parasites

As expected from basic probability theory and past work, generality increased the robustness of parasites to secondary extinction in the analytical model. Although the trajectory of a host-specific parasite was linear (+), generalist parasites (two and four hosts) were increasingly robust to secondary extinction, creating a concave down trajectory with biodiversity loss (figure 1).

#### (ii) Parasite life stages

As shown by other studies [28,33], the presence of multiple life stages in a parasite species greatly reduced the robustness of parasites to secondary extinction (figure 1). For instance, the concave up trajectory for parasite {1,1} is far below the diagonal for a single stage host-specific parasite (+), and opposite to the shape of the generalist parasite {2} with the same number of hosts. Adding generality for parasites with complex life cycles resulted in a diversity of curves (figure 1) that show how the opposing effects of generality and life stages affect the trajectory of a parasite species. If stages varied in generality, the stage with the minimum number of hosts tended to dominate the trajectory. For instance, parasite {1,10} had nearly the same trajectory as a single-stage host-specific parasite (+).

#### (iii) Differential extinction risks for hosts

Not surprisingly, a change in the probability of extinction for hosts relative to non-hosts altered the relationship between free-living and parasite richness (figure 2). If host and non-hosts had the same probability of being removed, the relationship was linear for a specialist parasite. If the host was more likely than non-hosts to be removed, the relationship was concave up. If the host was less likely to be removed than non-hosts, the relationship was concave down. Fits of models (2.4) and (2.5) are discussed in §3*b*.

### (b) Food-web models

#### (i) Effects of food-web topology

**Figure 3.**
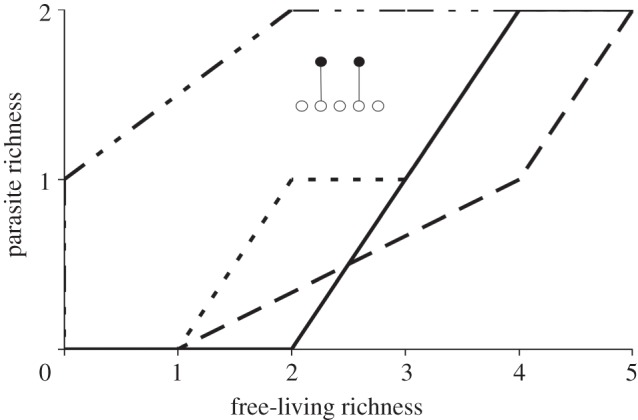
A food-web disassembly takes multiple paths. Plotted are four randomly selected trajectories for the disassembly of a system with five free-living and two parasite species, indicating how extinction order can lead to different relationships between free-living and parasite richness. Up to 5! trajectories are possible, though many trajectories would be redundant. Figure 4 shows the average trajectory (food web A) is a straight line. Feeding links connect parasitic (filled circles) and free-living (open circles) species.

**Figure 4.**
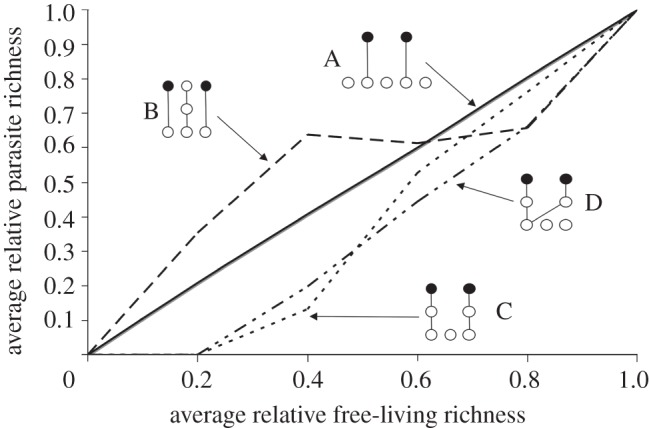
Food-web topology affects the relationship between parasite and free-living species richness. Feeding links connect parasite (filled circles) and free-living (open circles). Arrows point from a food web to its average disassembly path.

Figure 3 shows four of the 120 possible disassembly trajectories for a single food web of five free-living basal species and two parasite species that each uses a different host. In nature, it will be difficult to predict which trajectory will be taken unless extinction order is known. Averaging all trajectories would result in the straight line shown in figure 4, food web A, and this gives a general prediction for the trajectory of the parasite.

The topology of the food web altered the relationship between parasite and free-living species richness in ways not predictable from analytical models. In the illustrative example (figure 4), the two parasite species were always specialists and had a single stage, yet they had different average disassembly trajectories owing to simple differences in food-web topology. For food web A, all free-living species were independent and, because they were basal, all had no risk of secondary extinction. This fulfilled the assumptions of analytical models and led to the linear relationship between free-living and parasitic richness seen for a specialist parasite in figures 1 and 2. For food web B, non-hosts were more likely to suffer secondary extinctions than hosts. This increased the robustness of the parasites relative to free-living species. This was similar to the results of figure 2, except here the difference in extinction risk among free-living species was due solely to topological properties of the web. For food web C, hosts had a higher risk of extinction than non-hosts, leading to a concave up association between free-living and parasite richness (again consistent with figure 2). For food web D, the hosts shared a resource, so their fates were partly dependent. Their joint fate led to a conditional probability in the risk of secondary extinction for the parasite community, slightly changing the trajectory in comparison with food web C.

**Figure 5.**
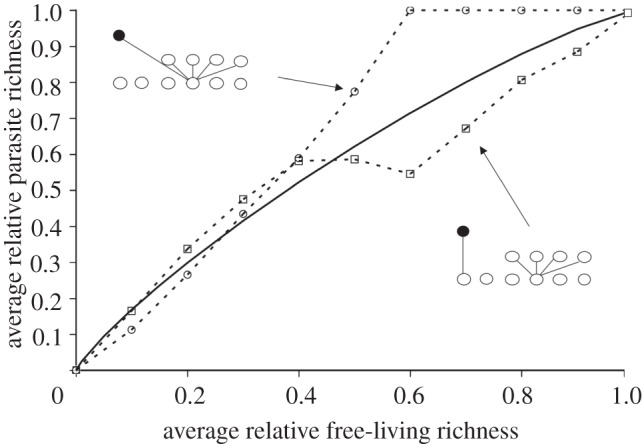
Food-web topology affects the relationship between parasite and free-living species richness for two simple food-web topologies that include one host-specific parasite, and ten free-living species (model (2.1*b*)). Feeding links connect parasitic (filled circles) and free-living (open circles). Arrows point from a food web to its average disassembly path. The solid line indicates the predicted path from the binomial model (which ignores food-web structure).

In the second illustrative example (figure 5), the parasite species were always specialists on a basal species, had a single stage, and were equally robust to secondary extinction, yet they had different disassembly trajectories owing to a change in a single link. For the network in the upper left of the figure, the parasite infected a host that supported many other specialist consumers. If the host went extinct, there were several simultaneous secondary extinctions, leading to a correlation between parasite extinction and biodiversity loss. This led to a strongly concave down relationship between parasite richness and free-living species richness. For the network in the lower right of figure 5, the parasite infected a host that supported no other consumers. When its host went extinct, no additional biodiversity was lost from the system and this led to an initially concave up relationship between host richness and parasite richness. The solid line is the analytical prediction from the binomial distribution (corrected for small sample size), which roughly splits the difference between the two networks. This illustrates how the analytical models cannot easily account for correlations in secondary extinctions between parasites and free-living species.

#### (ii) Empirical food webs

The empirical webs had 11–80 parasite species, which correlated positively with the 39–133 free-living species, although Otago Harbour had relatively few parasites (19) given its 126 free-living species. The average number of stages per parasite ranged from 1.5 to 2.3 and the average number of hosts per stage ranged from 1.9 to 7.7 (there was no association between these two variables).

**Figure 6.**
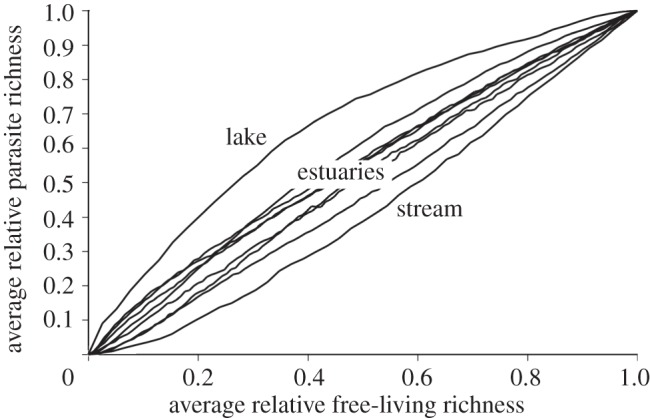
Associations between free-living species richness and parasite richness obtained from the disassembly of nine empirical food webs. The Arctic lake is the concave down curve, and the New Jersey stream is the concave up curve. The remaining quasi-linear paths were from seven estuaries (the Ythan River Estuary, Otago Harbour, Flensburg Estuary, Sylt Estuary, Carpinteria Salt Marsh, Estero de Punta Banda and Bahia San Quintín).

Individual parasite species' trajectories with biodiversity loss were split among concave down (38%), concave up (30%) and quasi-linear (28%) shapes (table 2). Only 3 per cent of parasite species had a sigmoid trajectory. However, the relative frequency of trajectory shapes varied among the food webs. For instance, most species in Muskingham Brook food web had concave down trajectories, whereas most of the parasites in the Lake Takvatn food web had concave up trajectories. Averaging the parasite species trajectories for each food web led to three relationships between parasite richness and free-living species richness (figure 6). The parasite community in the pelagic lake web (Takvatn) was the least sensitive to the loss of free-living species diversity, resulting in a concave down association between parasite and free-living species richness. This food web also had several single-stage generalist fungal parasites of phytoplankton that were robust to secondary extinction. The parasite community from the New Jersey stream was the least robust, owing to relatively low generality. The disassembly of this food web led to a concave up association between free-living biodiversity loss and parasite richness. The seven estuary food webs showed a quasi-linear relationship between parasite richness and free-living species richness. On average, the host relative loss order was 1.00, suggesting that secondary extinction risk for host species did not differ from non-hosts. Similarly, the average parasite relative loss order was 1.05, suggesting that parasites were not much more or less likely to go extinct than were free-living species (leading to the overall average linear relationship between free-living and parasite richness).

**Table 2.** Relative frequency distributions of the shape of the decline in the probability of a parasite species with declines in biodiversity for nine empirical food webs as calculated with a food-web disassembly model. Superscript letters indicate: E, estuary; S, stream; L, lake. BSQ, Bahía de San Quintín; CSM, Carpinteria Salt Marsh; EPB, Estero de Punta Banda.

web/shape	% distribution of parasite species trajectories			
	concave down	concave up	linear	sigmoid
BSQ^E^	35	40	17	8
CSM^E^	32	26	39	4
EPB^E^	21	28	43	8
Flensburg^E^	24	42	27	7
Muskingham^S^	69	8	23	0
Otago^E^	61	22	11	6
Sylt^E^	24	12	65	0
Takvatn^L^	18	73	9	0
Ythan^E^	62	19	19	0
average	38	30	28	3

Consistent with predictions from the analytical models, the relative robustness for each parasite species increased with an increasing number of hosts per stage and decreased with an increasing number of stages, and the standard deviation in the number of hosts per stage. Parasite relative robustness increased with the average robustness of the parasite's hosts, as measured by the relative loss order of hosts compared with non-hosts (table 3). Log-transforming hosts per stage dramatically improved the normality of the residual error and led to an *R*^2^ of 0.79 for the GLM.

**Table 3.** Results from a general linear model of parasite robustness to biodiversity loss for nine empirical food webs. Parasite robustness is measured as the average relative loss order of a parasite (during a series of food-web disassemblies) compared with the average free-living species in the same food web. The statistic for hosts per stage is the geometric mean (*R*^2^ = 0.79). Site was a random factor (variance component = 0.0013). *N* = 347 parasite species.

factor	estimate	s.e.	d.f.	d.f.den	*F*-ratio	*p*
intercept	1.178	0.030	1	68.3		<0.0001
stages	−0.253	0.013	1	341.6	364.66	<0.0001
s.d. hosts per stage	−0.086	0.015	1	336.2	31.54	<0.0001
host robustness	0.068	0.018	1	341.8	14.36	0.0002
log(hosts per stage)	0.342	0.012	1	341.6	811.55	<0.0001

The average robustness of parasites to secondary extinction, as measured by the average parasite relative loss order, could be explained by just two variables. Average parasite robustness to biodiversity loss increased with the average number of hosts per parasite stage and decreased with the average number of stages per parasite (table 4). On average, an increase in one parasite stage per parasite cancelled the robustness obtained by adding more than a dozen hosts.

**Table 4.** Results from a general linear model of average parasite robustness to biodiversity loss for nine empirical food webs. Average parasite robustness is measured as the average of relative loss orders (during a series of food-web disassemblies) of all parasites in a food webs compared with free-living species in that food web. The statistic for hosts per stage and stages per parasite is the geometric mean (*r*^2^ = 0.77; *N* = 9 food webs).

source, factor	estimate	s.e.	d.f.	SS	*F*-ratio	*p*
model			2	0.1	9.97	0.0124
error			6	0.0		
intercept	1.835	0.234	1			0.0002
hosts per stage	0.065	0.025	1	0.0	6.96	0.0386
stages per parasite	−0.524	0.126	1	0.1	17.34	0.0059

#### (iii) Differential extinction

**Figure 7.**
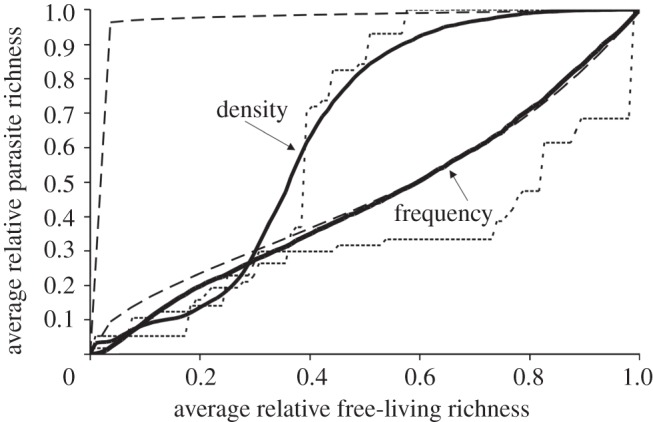
The relative extinction risk of hosts affects how parasite richness declines with free-living species richness in an empirical food web. Plotted are associations between free-living species richness and parasite richness obtained from the disassembly of the Carpinteria Salt Marsh food web under two different hypotheses about variation in the relative risk of extinction of free-living species: declining risk with biomass density (solid sigmoid line), and declining risk with the frequency a species was present in three estuaries (solid concave up line). Associated with each disassembly are estimates from the ‘variable host and parasite model’ (dashed lines, model (2.4*c*)) and the ‘variable host and parasite extinction order model’ (dotted lines, model (2.5*b*)).

As suggested by the analytical results (figure 2), adding variation to the primary extinction probabilities of the free-living species changed the relationship between parasite richness and free-living species richness (figure 7). When density was used as a measure of the resilience of free-living species to primary extinction, the trajectory was sigmoid, with parasite richness being robust to initial levels of biodiversity loss (relative loss order of 1.24), followed by a sharp decline in parasite richness as free-living species diversity dipped below 50 per cent. When species frequency was used as measure of resilience to secondary extinction (in my opinion, this is the more reasonable hypothesis), the parasite richness trajectory was concave up, indicating that parasites were more sensitive to biodiversity loss (relative loss order of 0.87) than would be expected if all hosts had the same risks of extinction (relative loss order of 1.08). This is consistent with the observation that several parasite species in this system depend on the extinction-prone snail *C. californica* [51].

#### (iv) Model performance

**Figure 8.**
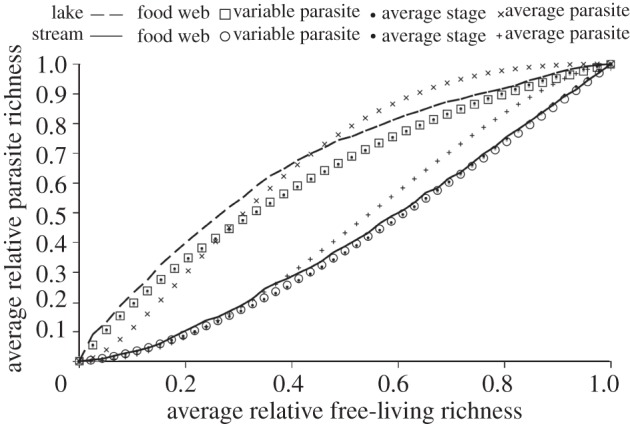
Relative fits of analytical models to trajectories created by food-web disassemblies. The (upper) concave down dashed line shows the disassembly for Lake Takvatn. The ‘variable parasite model’ (model (2.3*f*), square symbols) and ‘average stage model’ (model (2.3*d*), dot symbols) had similar trajectories that under estimated the disassembly model whereas the ‘average parasite model’ (model (2.3*c*), × symbols) initially over estimated and then under estimated. The lower concave up solid line shows the disassembly for Muskingham Brook. The ‘variable parasite model’ and ‘average stage model’ had similar trajectories that fit the disassembly model well. In comparison, the ‘average parasite model’ over estimated the disassembly trajectory.

The analytical models did not perfectly correspond to the food webs models (table 5; figures 7 and 8). Average absolute deviations ranged from 4 to 17 per cent. The ‘average parasite model’ (2.3*c*) fit relatively poorly. The simpler ‘average stage model’ (2.3*d*) fit as well as the more complex ‘variable parasite model’ (2.3*f*), indicating that the average stage model was a short cut worth taking. The performance of analytical models improved considerably at the community level, indicating that errors cancelled instead of magnified.

**Table 5.** Average absolute deviations between food-web models and analytical models for trajectories of parasite species and the parasite community in response to biodiversity loss. Smaller values indicate better fits to the food-web model. The ‘variable parasite model’ (models (2.3*e*,*f*)) is the most complex, followed by the ‘average stage model’ (model (2.3*d*)) and the ‘average parasite model’ (models (2.3*b*,*c*)) Superscripts indicate: E, estuary; S, stream; L, lake. BSQ, Bahía de San Quintín; CSM, Carpinteria Salt Marsh; EPB, Estero de Punta Banda. Note, for 500 iterations, the inherent absolute deviation among replicates of the food-web disassembly model was 0.02, suggesting a deviation near 0.02 was a good fit for an analytical model.

web/model	parasite species		parasite community		
	variable parasite	average stage	variable parasite	average stage	average parasite
BSQ^E^	0.07	0.08	0.02	0.02	0.22
CSM^E^	0.05	0.06	0.01	0.02	0.18
EPB^E^	0.06	0.07	0.01	0.02	0.14
Flensburg^E^	0.09	0.09	0.01	0.01	0.04
Muskingham^S^	0.04	0.04	0.01	0.02	0.05
Otago^E^	0.20	0.13	0.07	0.07	0.13
Sylt^E^	0.18	0.17	0.07	0.07	0.24
Takvatn^L^	0.09	0.09	0.05	0.05	0.06
Ythan^E^	0.09	0.09	0.01	0.01	0.05
average	0.10	0.09	0.03	0.03	0.12

The ‘variable host and parasite model’ (2.4*c*) was a good match for the food-web disassembly data in the case that extinction order was frequency-dependent. However, this model did not fit the density-dependent extinction order results well, primarily because of the high variance in density among the species (leading to several parasite species that could seemingly persist at low free-living biodiversity). Conversely, the ‘variable host and parasite extinction order model’ (2.5*b*) fit best when extinction order was density-dependent. The fit to the frequency-dependent extinction order was low because the extinction orders were not well defined, meaning that many alternative trajectories were equally likely.

## 4. Discussion

The average relationship between relative parasite richness and host richness could be linear, sigmoid, concave up or concave down and all empirical webs had a mix of parasites that varied in their robustness to biodiversity loss. This does not imply the lack of a general pattern. The overall average trajectory of these species was usually quasi-linear, suggesting a stronger effect of biodiversity loss on parasite richness than the concave down relationship previously estimated from models that considered only the number of hosts per parasite [8]. Moreover, the factors responsible for the shape were measurable.

Perhaps the most obvious factor affecting the risk to parasites of biodiversity loss was differential susceptibility of hosts to primary extinction. Assuming all free-living species have the same probability of primary extinction makes it easier to build analytical models. Changing this assumption (as I did for the analysis of Carpinteria Salt Marsh) greatly altered how parasite richness declined with biodiversity loss. Certainly, free-living species are not all equally susceptible to primary extinctions [41]. Humans target some species for fishing, hunting and other types of commercial exploitation. Demographic stochasticity and environmental variability should be more likely to affect large species with small population sizes [52]. In particular, species with small ranges or limited dispersal ability are less likely to be able to recolonize an area if they are locally extirpated [53]. Because parasites are not distributed randomly throughout food webs with respect to topology [39], they might not be randomly distributed with respect to the risk of hosts to primary extinction. Host species with small ranges should have fewer parasite species than species with large ranges, making parasite richness less sensitive to biodiversity loss [54]. By contrast, large free-living species tend to host more parasite species than do small free-living species [37,55,56]. Therefore, host susceptibility to primary extinction in a real system should be given a careful consideration when making specific predictions.

Generality decreased the sensitivity of parasites to losses in free-living species diversity because generalist parasites have multiple ways to complete their life cycles when their hosts go extinct. Unfortunately, knowing the average number of hosts per parasite in a community was not enough to estimate the relationship between parasite richness and host richness. Having multiple life stages increased the sensitivity of parasites to losses in free-living species diversity, because a parasite with a complex-life cycle needs more than one host to be present to persist, increasing the likelihood that some critical host will go extinct under scenarios of biodiversity loss. Life stages are an additional layer of host specificity that makes parasites extra-sensitive to secondary extinction [28]. As indicated in the statistical models, the average number of hosts per life stage is meaningful only in the context of the variation in the number of hosts per life stage. A host-specific life stage can make a parasite susceptible to secondary extinction even if other life stages can use many hosts. The s.d. of hosts per stage was, therefore, a useful predictive variable for parasite robustness in analytical models.

Food-web topology was the hardest factor to account for with analytical models because food webs lead to interactions among species, resulting in conditional probabilities that are difficult to estimate analytically. The removal of one host can lead to the loss of many parasites, and the removal of an important basal species could lead to the loss of many hosts simultaneously. Although it is difficult to predict the net effect of food-web topology on parasites, the results of this study suggest that analytical models without information on the topology of free-living species can often adequately predict the general shape of the empirical relationship between free-living and parasitic richness. Although host robustness to secondary extinction was useful in explaining the robustness of a particular parasite to secondary extinction, the average robustness of the host community (weighted by parasite richness) did not help predict the average robustness of the parasite community. It is difficult to explain why the effect of host robustness did not scale up to the community level. Consistent with past findings [33], hosts were not, on average, more or less likely to suffer secondary extinctions than were non-hosts based on their position in a food-web topology. Averages might obscure important, but variable, contributions at the species level.

Although I focused on biodiversity loss, one could also consider how introduced species or biodiversity restoration could affect the relationship between parasite and free-living species diversity. If, as is common, introduced species leave their parasites behind, parasite diversity will not respond strongly to increases in free-living species diversity due to invasion. If the parasite-poor invaders out compete native species with many parasites, the result could be a decrease in the parasite richness of the system even as new free-living species are added [34,51]. Otherwise, new free-living species can bring parasites into a system of resident hosts that lack prior exposure. In these cases, the new parasite diversity could reduce free-living species richness through disease-driven extinctions, though such events are rare [57]. Therefore, species additions could lead to a negative association between free-living and parasite richness. There can be a positive relationship between invasive parasites and hosts if invaders do not escape natural enemies. The human-mediated addition of two regionally common fish to a species-poor lake appears to have facilitated the addition of five parasite species [58]. Similarly, if restoration of native biodiversity occurs, we should see an increase in both parasite and free-living biodiversity. For instance, the diversity of trematodes in estuarine snails increased steadily over 6 years after a habitat restoration project, presumably because the restoration succeeded in creating habitat for the various invertebrates, fishes and birds that the trematodes required to complete their life cycles [14]. However, not all restoration efforts will succeed in attracting parasites. In a heathland restoration, the lack of parasites to fully recolonize the region suggested that the effort had failed to recreate the complex trophic interactions found in natural habitats [59].

The alternative perspective that biodiversity loss will increase infectious disease comes from cases of the dilution effect and host compensation. Dilution effects occur when some host species interfere with parasite transmission. The removal of interfering hosts (but not those that are required for transmission) can lead to an increase in the prevalence of certain infectious diseases such as Lyme [4]. The net outcome in prevalence for a particular parasite depends on whether the required hosts or the interfering free-living species are more likely to suffer extinctions. Host compensation can result if some hosts become abundant due to release from predators or competition, leading to more efficient transmission of infectious diseases [60]. The importance of host compensation depends on the strength of trophic cascades and the relative abundances of impacted versus released hosts. Although dilution and compensation are possible results of biodiversity loss, there is no logical reason to expect that they will be the rule or overshadow secondary extinctions.

The strong association between parasite richness and host richness suggests that parasites can be positive indicators of free-living species richness [13,15,61]. Specifically, a high diversity of parasites indicates a complex and functioning set of interacting free-living species. For example, the abundance of parasitoid insects increases remarkably with native plant diversity in the Azores [62]. Some parasites will be more suitable indicators of free-living species diversity than others. The best source of indicative parasites would be an easy to sample, abundant host species that has a high diversity of parasites with complex life cycles. In aquatic systems, ideal parasite communities for monitoring are found in fishes [17,63–65] and snails [22,24,66,67].

Dynamical models [42,68] are a tool that could help answer some remaining questions. Such models could allow parasites to impact their hosts, and non-competent hosts to impact parasites, leading to more complex relationships between parasite and free-living species diversity. Four possibilities might emerge from dynamical models. If parasites have density-dependent effects on a guild of competitors, they might prevent competitive exclusion and promote diversity [69]. Alternatively, if parasites are generalists and vary in their impact on hosts, they could drive intolerant host species extinct [70]. If some hosts interfere with parasite transmission, their addition to a system could reduce parasite diversity via the dilution effect [4]. Finally, because parasites might go extinct before their hosts [71], parasites might respond even more strongly to biodiversity loss than seen in topological models. Unfortunately, there is not sufficient empirical data to build dynamical models with parasites, so this approach is limited to hypothetical systems for now.

In conclusion, parasite richness declines as free-living biodiversity is lost, resulting in a positive association between parasite richness and free-living species richness. As is the case for free-living species, parasite generality buffers parasites to host losses, whereas complex life cycles add an extra set of resource (host) requirements that reduce parasite robustness to secondary extinctions. Food-web topology leads to conditional probabilities that can complicate how parasite richness relates to free-living species richness. On average, empirical webs showed a quasi-linear decline in relative parasite richness with relative free-living species richness. As a result, parasites are sensitive to free-living species diversity and some can be useful as bioindicators of ecosystem degradation and recovery. Although I focus on parasites, these results should be applicable to most affiliate species (mutuals and commensals).

## References

[1] Harvell C. D. Mitchell C. E. Ward J. R. Altizer S. Dobson A. P. Ostfeld R. S. Samuel M. D. 2002 Climate warming and disease risks for terrestrial and marine biota. Science 296, 2158–2162 10.1126/science.1063699 (doi:10.1126/science.1063699) 12077394

[2] Keesing F. 2010 Impacts of biodiversity on the emergence and transmission of infectious diseases. Nature 468, 647–652 10.1038/nature09575 (doi:10.1038/nature09575) 21124449 PMC7094913

[3] Swaddle J. P. Calos S. E. 2008 Increased avian diversity is associated with lower incidence of human West Nile infection: observation of the dilution effect. PLoS ONE 3, e2488 10.1371/journal.pone.0002488 (doi:10.1371/journal.pone.0002488) 18575599 PMC2427181

[4] Keesing F. Holt R. D. Ostfeld R. S. 2006 Effects of species diversity on disease risk. Ecol. Lett. 9, 485–498 10.1111/j.1461-0248.2006.00885.x (doi:10.1111/j.1461-0248.2006.00885.x) 16623733

[5] Pongsiri M. J. Roman J. Ezenwa V. O. Goldberg T. L. Koren H. S. Newbold S. C. Ostfeld R. S. Pattanayak S. K. Salkeld D. J. 2009 Biodiversity loss affects global disease ecology. Bioscience 59, 945–954 10.1525/bio.2009.59.11.6 (doi:10.1525/bio.2009.59.11.6)

[6] Dobson A. P. Lafferty K. D. Kuris A. M. Hechinger R. F. Jetz W. 2008 Homage to Linnaeus: how many parasites? How many hosts? Proc. Natl Acad. Sci. USA 105, 11 482–11 489 10.1073/pnas.0803232105 (doi:10.1073/pnas.0803232105) 18695218 PMC2556407

[7] Dunn R. R. Harris N. C. Colwell R. K. Koh L. P. Sodhi N. S. 2009 The sixth mass coextinction: are most endangered species parasites and mutualists? Proc. R. Soc. B 276, 3037–3045 10.1098/rspb.2009.0413 (doi:10.1098/rspb.2009.0413) PMC2817118 19474041

[8] Koh L. P. Dunn R. R. Sodhi N. S. Colwell R. K. Proctor H. C. Smith V. S. 2004 Species coextinctions and the biodiversity crisis. Science 305, 1632–1634 10.1126/science.1101101 (doi:10.1126/science.1101101) 15361627

[9] Poulin R. Morand S. 1997 Parasite body size distributions: interpreting patterns of skewness. Int. J. Parasitol. 27, 959–964 10.1016/S0020-7519(97)00055-6 (doi:10.1016/S0020-7519(97)00055-6) 9292313

[10] Sprent J. F. A. 1992 Parasites lost. Int. J. Parasitol. 22, 139–151 10.1016/0020-7519(92)90095-3 (doi:10.1016/0020-7519(92)90095-3) 1587677

[11] Dobson A. P. May R. M. 1987 The effects of parasites on fish populations - theoretical aspects. Int. J. Parasitol. 17, 363–370 10.1016/0020-7519(87)90111-1 (doi:10.1016/0020-7519(87)90111-1) 3294650

[12] Hechinger R. F. Lafferty K. D. Huspeni T. C. Brooks A. Kuris A. M. 2007 Can parasites be indicators of free-living diversity? Relationships between species richness and the abundance of larval trematodes and of local fishes and benthos. Oecologia 151, 82–92 10.1007/s00442-006-0568-z (doi:10.1007/s00442-006-0568-z) 17024376

[13] Hudson P. J. Dobson A. P. Lafferty K. D. 2006 Parasites and ecological systems: is a healthy system one with many parasites? Trends Ecol. Evol. 21, 381–385 10.1016/j.tree.2006.04.007 (doi:10.1016/j.tree.2006.04.007) 16713014

[14] Huspeni T. C. Lafferty K. D. 2004 Using larval trematodes that parasitize snails to evaluate a salt-marsh restoration project. Ecol. Appl. 14, 795–804 10.1890/01-5346 (doi:10.1890/01-5346)

[15] Lafferty K. D. 1997 Environmental parasitology: what can parasites tell us about human impacts on the environment? Parasitol. Today 13, 251–255 10.1016/S0169-4758(97)01072-7 (doi:10.1016/S0169-4758(97)01072-7) 15275061

[16] Lafferty K. D. Kuris A. M. 1999 How environmental stress affects the impacts of parasites? Limnol. Oceanogr. 44, 925–931 10.4319/lo.1999.44.3_part_2.0925 (doi:10.4319/lo.1999.44.3_part_2.0925)

[17] Wood C. L. Lafferty K. D. Micheli F. 2010 Fishing out marine parasites? Impacts of fishing on rates of parasitism in the ocean. Ecol. Lett. 13, 761–775 10.1111/j.1461-0248.2010.01467.x (doi:10.1111/j.1461-0248.2010.01467.x) 20412277

[18] Byers J. E. Altman I. Grosse A. M. Huspeni T. C. Maerz J. C. 2011 Using parasitic trematode larvae to quantify an elusive vertebrate host. Conserv. Biol. 25, 85–93 10.1111/j.1523-1739.2010.01583.x (doi:10.1111/j.1523-1739.2010.01583.x) 21029163

[19] Guernier V. Hochberg M. E. Guegan J. F. O. 2004 Ecology drives the worldwide distribution of human diseases. PLoS Biol. 2, 740–746 10.1371/journal.pbio.0020141 (doi:10.1371/journal.pbio.0020141) PMC423130 15208708

[20] Dunn R. R. Davies T. J. Harris N. C. Gavin M. C. 2010 Global drivers of human pathogen richness and prevalence. Proc. R. Soc. B 277, 2587–2595 10.1098/rspb.2010.0340 (doi:10.1098/rspb.2010.0340) PMC2982038 20392728

[21] Harris N. C. Dunn R. R. 2010 Using host associations to predict spatial patterns in the species richness of the parasites of North American carnivores. Ecol. Lett. 13, 1411–1418 10.1111/j.1461-0248.2010.01527.x (doi:10.1111/j.1461-0248.2010.01527.x) 20875037

[22] Hechinger R. F. Lafferty K. D. 2005 Host diversity begets parasite diversity: bird final hosts and trematodes in snail intermediate hosts. Proc. R. Soc. B 272, 1059–1066 10.1098/rspb.2005.3070 (doi:10.1098/rspb.2005.3070) PMC1599879 16024365

[23] Lafferty K. D. Dunham E. J. 2005 Trematodes in snails near raccoon latrines suggest a final host role for this mammal in California salt marshes. J. Parasitol. 91, 474–476 10.1645/GE-390R1 (doi:10.1645/GE-390R1) 15986632

[24] Whitney K. L. Hechinger R. F. Kuris A. M. Lafferty K. D. 2007 Endangered light-footed clapper rail affects parasite community structure in coastal wetlands. Ecol. Appl. 17, 1694–1702 10.1890/06-1325.1 (doi:10.1890/06-1325.1) 17913133

[25] Lafferty K. D. Shaw J. C. Kuris A. M. 2008 Reef fishes have higher parasite richness at unfished Palmyra Atoll compared to fished Kiritimati Island. EcoHealth 5, 338–345 10.1007/s10393-008-0196-7 (doi:10.1007/s10393-008-0196-7) 18846315

[26] Loot G. Aldana M. Navarrete S. A. 2005 Effects of human exclusion on parasitism in intertidal food webs of central Chile. Conserv. Biol. 19, 203–212 10.1111/j.1523-1739.2005.00396.x (doi:10.1111/j.1523-1739.2005.00396.x)

[27] McIntyre P. B. Michel E. France K. Rivers A. Hakizimana P. Cohen A. S. 2005 Individual- and assemblage-level effects of anthropogenic sedimentation on snails in Lake Tanganyika. Conserv. Biol. 19, 171–181 10.1111/j.1523-1739.2005.00456.x (doi:10.1111/j.1523-1739.2005.00456.x)

[28] Rudolf V. Lafferty K. D. 2011 Stage structure alters how complexity affects stability of ecological networks. Ecol. Lett. 14, 75–79 10.1111/j.1461-0248.2010.01558.x (doi:10.1111/j.1461-0248.2010.01558.x) 21114747

[29] Dunne J. A. Williams R. J. 2009 Cascading extinctions and community collapse in model food webs. Phil. Trans. R. Soc. B 364, 1711–1723 10.1098/rstb.2008.0219 (doi:10.1098/rstb.2008.0219) 19451122 PMC2685420

[30] Dunne J. A. Williams R. J. Martinez N. D. 2002 Food-web structure and network theory: the role of connectance and size. Proc. Natl Acad. Sci. USA 99, 12 917–12 922 10.1073/pnas.192407699 (doi:10.1073/pnas.192407699) PMC130560 12235364

[31] Solé R. V. Montoya J. M. 2001 Complexity and fragility in ecological networks. Proc. R. Soc. Lond. B 268, 2039–2045 10.1098/rspb.2001.1767 (doi:10.1098/rspb.2001.1767) PMC1088846 11571051

[32] Price R. D. Clayton D. H. Adams R. J. 2000 Pigeon lice down under: taxonomy of Australian Campanulotes (Phthiraptera: Philopteridae), with a description of *C. durdeni* n. sp. J. Parasitol. 86, 948–950 10.1645/0022-3395(2000)086[0948:PLDUTO]2.0.CO;2 (doi:10.1645/0022-3395(2000)086[0948:PLDUTO]2.0.CO;2) 11128516

[33] Chen H.-W. Shao K. T. Liu C. W.-J. Lin W.-H. Liu W. C. 2011 The reduction of food web robustness by parasitism: fact and artefact. Int. J. Parasitol. 4, 627–634 10.1016/j.ijpara.2010.12.013 (doi:10.1016/j.ijpara.2010.12.013) 21296081

[34] Lafferty K. D. Kuris A. M. 2009 Parasites reduce food web robustness because they are sensitive to secondary extinction as illustrated by an invasive estuarine snail. Phil. Trans. R. Soc. B 364, 1659–1663 10.1098/rstb.2008.0220 (doi:10.1098/rstb.2008.0220) 19451117 PMC2685421

[35] Altizer S. Nunn C. L. Lindenfors P. 2007 Do threatened hosts have fewer parasites? A comparative study in primates. J. Anim. Ecol. 76, 304–314 10.1111/j.1365-2656.2007.01214.x (doi:10.1111/j.1365-2656.2007.01214.x) 17302838

[36] Purvis A. Gittleman J. L. Cowlishaw G. Mace G. M. 2000 Predicting extinction risk in declining species. Proc. R. Soc. Lond. B 267, 1947–1952 10.1098/rspb.2000.1234 (doi:10.1098/rspb.2000.1234) PMC1690772 11075706

[37] Vitone N. D. Altizer S. Nunn C. L. 2004 Body size, diet and sociality influence the species richness of parasitic worms in anthropoid primates. Evol. Ecol. Res. 6, 183–199

[38] Lafferty K. D. Dobson A. P. Kuris A. M. 2006 Parasites dominate food web links. Proc. Natl Acad. Sci. USA 103, 11 211–11 216 10.1073/pnas.0604755103 (doi:10.1073/pnas.0604755103) PMC1544067 16844774

[39] Chen H. W. Liu W. C. Davis A. J. Jordan F. Hwang M. J. Shao K. T. 2008 Network position of hosts in food webs and their parasite diversity. Oikos 117, 1847–1855 10.1111/j.1600-0706.2008.16607.x (doi:10.1111/j.1600-0706.2008.16607.x)

[40] Gerber L. R. Lafferty K. D. McCallum H. I. Sabo J. L. Dobson A. P. 2005 Exposing extinction risk analysis to pathogens: is disease just another form of density dependence? Ecol. Appl. 15, 1402–1414 10.1890/04-0880 (doi:10.1890/04-0880)

[41] Srinivasan U. T. Dunne J. A. Harte J. Martinez N. D. 2007 Response of complex food webs to realistic extinction sequences. Ecology 88, 671–682 10.1890/06-0971 (doi:10.1890/06-0971) 17503595

[42] Lafferty K. D. Dunne J. A. 2010 Stochastic ecological network occupancy (SENO) models: a new tool for modeling ecological networks across spatial scales. Theor. Ecol. 3, 123–135 10.1007/s12080-010-0082-0 (doi:10.1007/s12080-010-0082-0)

[43] Hernandez A. D. Sukhdeo M. V. K. 2008 Parasites alter the topology of a stream food web across seasons. Oecologia 156, 613–624 10.1007/s00442-008-0999-9 (doi:10.1007/s00442-008-0999-9) 18305960

[44] Amundsen P. A. Lafferty K. D. Knudsen R. Primicerio R. Klemetsen A. Kuris A. M. 2009 Food web topology and parasites in the pelagic zone of a subarctic lake. J. Anim. Ecol. 78, 563–572 10.1111/j.1365-2656.2008.01518.x (doi:10.1111/j.1365-2656.2008.01518.x) 19175443

[45] Huxham M. Raffaelli D. 1995 Parasites and food-web patterns. J. Anim. Ecol. 64, 168–176 10.2307/5752 (doi:10.2307/5752)

[46] Mouritsen K. N. Poulin R. McLaughlin J. P. Thieltges D. W. 2011 Food web including metazoan parasites for an intertidal ecosystem in New Zealand. Ecology 92, 2006 10.1890/11-0371.1 (doi:10.1890/11-0371.1)

[47] Thieltges D. W. Reise K. Mouritsen K. N. McLaughlin J. P. Poulin R. 2011 Food web including metazoan parasites for a tidal basin in Germany/Denmark. Ecology 92, 2005 10.1890/11-0351.1 (doi:10.1890/11-0351.1)

[48] Zander C. D. Josten N. Detloff K. C. Poulin R. McLaughlin J. P. Thieltges D. W. 2011 Food web including metazoan parasites for a brackish shallow water ecosystem in Germany and Denmark. Ecology 92, 2007 10.1890/11-0374.1 (doi:10.1890/11-0374.1)

[49] Hechinger R. F. 2011 Food webs including parasites, biomass, body sizes, and life stages for three California/Baja California estuaries. Ecology 92, 791–792 10.1890/10-1383.1 (doi:10.1890/10-1383.1)

[50] Byers J. E. 1999 The distribution of an introduced mollusc and its role in the long-term demise of a native confamilial species. Biol. Invasions 1, 339–352 10.1023/A:1010038001768 (doi:10.1023/A:1010038001768)

[51] Torchin M. E. Byers J. E. Huspeni T. C. 2005 Differential parasitism of native and introduced snails: replacement of a parasite fauna. Biol. Invasions 7, 885–894 10.1007/s10530-004-2967-6 (doi:10.1007/s10530-004-2967-6)

[52] Cardillo M. Mace G. M. Jones K. E. Bielby J. Bininda-Emonds O. R. P. Sechrest W. Orme C. D. L. Purvis A. 2005 Multiple causes of high extinction risk in large mammal species. Science 309, 1239–1241 10.1126/science.1116030 (doi:10.1126/science.1116030) 16037416

[53] Lee T. M. Jetz W. 2011 Range size is a common factor associated with risk of extinction in a range of organisms. Proc. R. Soc. B 278, 1329–1338 10.1098/rspb.2010.1877 (doi:10.1098/rspb.2010.1877)

[54] Lindenfors P. Nunn C. L. Jones K. E. Cunningham A. A. Sechrest W. Gittleman J. L. 2007 Parasite species richness in carnivores: effects of host body mass, latitude, geographical range and population density. Glob. Ecol. Biogeogr. 16, 496–509 10.1111/j.1466-8238.2006.00301.x (doi:10.1111/j.1466-8238.2006.00301.x)

[55] Gregory R. D. Keymer A. E. Harvey P. H. 1996 Helminth parasite richness among vertebrates. Biodivers. Conserv. 5, 985–997 10.1007/BF00054416 (doi:10.1007/BF00054416)

[56] Poulin R. 1995 Phylogeny, ecology and the richness of parasite communities in vertebrates. Ecol. Monogr. 65, 283–302 10.2307/2937061 (doi:10.2307/2937061)

[57] Smith K. E. Sax D. E. Lafferty K. D. 2006 Evidence for the role of infectious disease in species extinction and endangerment. Conserv. Biol. 20, 1349–1357 10.1111/j.1523-1739.2006.00524.x (doi:10.1111/j.1523-1739.2006.00524.x) 17002752

[58] Amundsen P. A. Lafferty K. D. Knudsen R. Primicerio R. Kristoffersen R. Klemetsen A. Kuris A. M. In press New parasites and predators follow the introduction of two fish species to a subarctic lake: implications for food-web structure and functioning. Oecologia. 10.1007/s00442-012-2461-2 PMC3612402 23053223

[59] Henson K. S. E. Craze P. G. Memmott J. 2009 The restoration of parasites, parasitoids, and pathogens to heathland communities. Ecology 90, 1840–1851 10.1890/07-2108.1 (doi:10.1890/07-2108.1) 19694133

[60] Lafferty K. D. 2004 Fishing for lobsters indirectly increases epidemics in sea urchins. Ecol. Appl. 14, 1566–1573 10.1890/03-5088 (doi:10.1890/03-5088)

[61] Marcogliese D. J. 2005 Parasites of the superorganism: are they indicators of ecosystem health? Int. J. Parasitol. 35, 705–716 10.1016/j.ijpara.2005.01.015 (doi:10.1016/j.ijpara.2005.01.015) 15925594

[62] Heleno R. H. Ceia R. S. Ramos J. A. Memmott J. 2009 Effects of alien plants on insect abundance and biomass: a food-web approach. Conserv. Biol. 23, 410–419 10.1111/j.1523-1739.2008.01129.x (doi:10.1111/j.1523-1739.2008.01129.x) 19128322

[63] Marcogliese D. Ball M. Lankester M. W. 2001 Potential impacts of clearcutting on parasites of minnows in small boreal lakes. Folia Parasitol. 48, 269–274 11817450 10.14411/fp.2001.045

[64] Marcogliese D. Gendron A. Plante C. Fournier M. Cyr D. 2006 Parasites of spottail shiners (*Notropis hudsonius*) in the St. Lawrence River: effects of municipal effluents and habitat. Can. J. Zool. 84, 1461–1481 10.1139/z06-088 (doi:10.1139/z06-088)

[65] Marcogliese D. J. Cone D. K. 1996 On the distribution and abundance of eel parasites in Nova Scotia: influence of pH. J. Parasitol. 82, 389–399 10.2307/3284074 (doi:10.2307/3284074) 8636841

[66] Huspeni T. C. Hechinger R. F. Lafferty K. D. 2005 Trematode parasites as estuarine indicators: opportunities, applications and comparisons with conventional community approaches. In Estuarine indicators (ed. Bortone S. A.), pp. 297–314 Boca Raton, FL: CRC Press

[67] Lafferty K. D. Hechinger R. F. Lorda J. Soler L. 2005 Trematodes associated with mangrove habitat in Puerto Rican salt marshes. J. Parasitol. 91, 697–699 10.1645/GE-427R (doi:10.1645/GE-427R) 16108572

[68] Williams R. J. Brose U. Martinez N. D. 2007 Homage to Yodzis and Innes 1992: scaling up feeding-based population dynamics to complex ecological networks. In From energetics to ecosystems: the dynamics and structure of ecological systems (eds Rooney N. McCann K. S. Noakes D. L. G.), pp. 37–52 Berlin, Germany: Springer

[69] Clay K. Reinhart K. Rudgers J. Tintjer Tammy Koslow J. Flory S. L. 2008 Red queen communities. In Infectious disease ecology: effects of disease on ecosystems and of ecosystems on disease (eds Ostfeld R. Keesing F. Eviner V.), pp. 145–178 Millbrook, NY: Institute for Ecosystem Studies

[70] Tompkins D. M. Greenman J. V. Robertson P. A. Hudson P. J. 2000 The role of shared parasites in the exclusion of wildlife hosts: *Heterakis gallinarum* in the ring-necked pheasant and the grey partridge. J. Anim. Ecol. 69, 829–840 10.1046/j.1365-2656.2000.00439.x (doi:10.1046/j.1365-2656.2000.00439.x) 29313999

[71] Lloyd-Smith J. O. Cross P. C. Briggs C. J. Daugherty M. Getz W. M. Latto J. Sanchez M. S. Smith A. B. Swei A. 2005 Should we expect population thresholds for wildlife disease? Trends Ecol. Evol. 20, 511–519 10.1016/j.tree.2005.07.004 (doi:10.1016/j.tree.2005.07.004) 16701428

